# Comparison of the cost of illness of primary liver cancer between Japan and Taiwan

**DOI:** 10.1186/s13561-020-00296-7

**Published:** 2020-12-05

**Authors:** Yinghui Wu, Kunichika Matsumoto, Ya-Mei Chen, Yu-Chi Tung, Tzu-Ying Chiu, Tomonori Hasegawa

**Affiliations:** 1 School of Nursing, Shanghai Jiao Tong University, Shanghai, China; 2 Department of Social Medicine, Toho University School of Medicine, 5-21-16 Omori-Nishi, Ota-ku, Tokyo, 143-8540 Japan; 3 Institute of Health Policy & Management, College of Public Health, National Taiwan University, Taipei, Taiwan; 4 Graduate Institute of Long-term Care, Tzu Chi University of Science and Technology, Hualien, Taiwan

**Keywords:** Cost of illness, Health policy, Liver neoplasms, Medical economics, Public health

## Abstract

**Background:**

Primary liver cancer (PLC) is the fifth and second leading cause of death in Japan and Taiwan, respectively. The aim of this study was to compare the economic burden of PLC between the two countries using the cost of illness (COI) method and identify the key factors causing the different trends in the economic burdens of PLC.

**Materials and methods:**

We calculated the COI every 3 years using governmental statistics of both countries (1996–2014 data for Japan and 2002–2014 data for Taiwan). The COI was calculated by summing the direct costs, morbidity costs, and mortality costs. We compared the COIs of PLC in both countries at the USD-based cost. The average exchange rate during the targeted years was used to remove the impact of foreign exchange volatility.

**Results:**

From 1996 to 2014, the COI exhibited downward and upward trends in Japan and Taiwan, respectively. In Japan, the COI in 2014 was 0.70 times the value in 1996, and in Taiwan, the COI in 2014 was 1.16 times greater than that in 1996. The mortality cost was the greatest contributor in both countries and had the largest contribution ratio to the COI increase in Japan. However, the direct cost in Taiwan had the largest contribution ratio to the COI decrease.

**Conclusions:**

To date, the COI of PLC in Japan has continuously decreased, whereas that in Taiwan has increased. Previous health policies and technological developments are thought to have accelerated the COI decrease in Japan and are expected to change the trend of COI of PLC, even in Taiwan.

## Background

Primary liver cancer (PLC; International Disease Classification 10 code C22) is an important disease in Asia. PLC is the fifth leading cause of death (the fourth among males and the sixth among females) in Japan [1] and the second leading cause of death (the second among both males and females) in Taiwan [2]. Most cases of PLC are caused by chronic hepatitis B virus (HBV) or hepatitis C virus (HCV) infection [3–5]. PLC caused by chronic HCV infection is predominant in Japan, whereas HBV infection is the more predominant cause of PLC in Taiwan [6, 7].

Although PLC is an important cause of death, to date, few studies have attempted to estimate the total economic burden of PLC or other liver diseases in both countries. Several studies have assessed the economic burden of liver diseases in East Asian countries [8–11]; however, only our studies have investigated the burden in Japan [12, 13]. Moreover, no studies have examined the burden of liver diseases in Taiwan.

In this study, we calculated the economic burden of PLC in Japan and Taiwan over time using the cost of illness (COI) method, which included direct costs (DC) and indirect costs (IC), including opportunity cost due to disease and death. This study also focused on key factors leading to different economic burden of PLC trends between the two countries.

## Methods

In this study, the COI method proposed by Rice et al. was used to investigate the economic burden of PLC in Japan and Taiwan [14–20]. To survey the trend of the COI, available governmental data were used (1996–2014 data for Japan and 2002–2014 data for Taiwan). For the Japanese COI, we used the same data set that was used in a previous study [21] and re-calculated the COI for comparison with the Taiwanese COI.

The COI includes both direct costs (DC) and indirect costs (IC), and the IC include morbidity costs (MbC) and mortality costs (MtC). The COI was calculated using the following equation:

$$ \mathrm{COI}=\mathrm{DC}+\mathrm{MbC}+\mathrm{MtC} $$

where DC refers to the medical costs that arise directly as a result of the disease, such as the costs of treatment, hospitalization, testing and drugs. We calculated the annual medical costs in Japan based on the total medical expenses using the Survey of National Medical Care Insurance Services [22]. For the Taiwanese annual medical costs, we first calculated the ratio of medical benefit payments for PLC to the total medical benefit payments using the National Health Insurance Annual Statistics [23] and calculated the direct cost of PLC by multiplying the ratio and the total medical expenses in Taiwan.

MbC refers to the loss of opportunity costs resulting from hospitalization and visits to the hospital. MbC was calculated using the following equation:

$$ \mathrm{MbC}=\mathrm{TO}{\mathrm{V}}_{\mathrm{y}}\times \frac{\mathrm{L}{\mathrm{V}}_{\mathrm{d}}}{2}+\mathrm{THD}\times \mathrm{L}{\mathrm{V}}_{\mathrm{d}} $$

where TOVy refers to the total person-days of outpatient visits, LVd refers to the 1-day labour value per person, and THD refers to the total person-days of hospitalization. The TOVy and THD were calculated according to gender and 5-year age groups in Japan based on the patient survey [24], and those in Taiwan were calculated based on the National Health Insurance Annual Statistics [25]. The labour values in Japan were calculated according to 5-year age groups using the Basic Survey on Wage Structure, Labor Force Survey, and Estimates of Monetary Valuation of Unpaid Work [26–28], and the labour values in Taiwan were calculated using the Manpower Survey [29]. There are no data regarding unpaid work in Taiwan. Accordingly, unpaid work was estimated by multiplying the Taiwanese formal labour value by the Japanese ratio of monetary value of unpaid work to the formal labour value. LVd and THD were calculated as follows:

$$ \mathrm{L}{\mathrm{V}}_{\mathrm{d}}=\frac{\left({\mathrm{I}}_{\mathrm{y}}+\mathrm{UL}{\mathrm{V}}_{\mathrm{y}}\right)}{365} $$

$$ \mathrm{THD}=\mathrm{H}{\mathrm{P}}_{\mathrm{y}}\times \mathrm{ALOS} $$

where Iy refers to the annual income per person, ULVy refers to the annual monetary valuation of unpaid work per person, HPy refers to the annual number of hospitalized patients, and ALOS refers to the average length of stay.

MtC was measured as the loss of human capital (human capital method), which was calculated using the following equation:

$$ \mathrm{MtC}=\mathrm{N}{\mathrm{D}}_{\mathrm{y}}\times \mathrm{LVl} $$

where NDy refers to the number of deaths, and LVl refers to the lifetime labour value per person. We calculated LVl by summing the income that could have been earned in the future if death had not occurred. The future labour value was adjusted to a present value using a 2% discount rate because 2% is recommended by Japanese guidelines for economic evaluation of healthcare technologies [30]. For these calculations, we used the Basic Survey on Wage Structure, Labor Force Survey, and Estimates of Monetary Valuation of Unpaid Work [26–28] for Japan and the Manpower Survey [29] for Taiwan. For NDy, we used the number of deaths due to PLC according to gender and 5-year age groups from Vital Statistics [1] for Japan and the Cause of Death Statistics [31] for Taiwan.

The COIs of PLC in both countries were compared at the USD-based cost. The average exchange rate during the targeted years was used to adjust for the impact of foreign exchange volatility.

## Results

Table 1 shows the trends in the COI and health-related indicators in both countries. In 2014, the COI in Japan was calculated as 607.2 billion Japanese yen (JPY) (≒5.69 billion USD), and the COI in Taiwan was calculated as 25.8 billion New Taiwan dollars (NTD) (≒0.80 billion USD). The contributions of DC, MbC, and MtC to the COI were 131.6 billion JPY (27.1%), 18.5 billion JPY (3.0%), and 457.1 billion JPY (75.3%) in Japan and 8507.5 million NTD (33.0%), 367.9 million NTD (1.4%), and 16,879.4 million NTD (65.5%) in Taiwan, respectively. MtC was the greatest contributor in both countries. The COI continuously decreased from 1996 to 2014 with an annual percent change (APC) of − 2.0%, resulting in a 0.70-fold decrease in Japan. However, the COI continuously increased from 2002 to 2014 with an APC of 1.3%, resulting in a 1.15-fold increase in Taiwan. In Japan, DC increased until 2002 and gradually decreased thereafter; MbC decreased since 1999; and MtC, which accounted for more than 70% of the COI, decreased since 2002. The contribution ratio of MtC to the total COI decrease was 106.9%. In Taiwan, DC continuously increased until 2014; MbC increased until 2011 and decreased thereafter; and MtC slightly decreased. DC had the highest contribution ratio to the total COI increase of 116.5%.

**Table 1:** The time trend of cost of illness (COI) of liver cancer

JAPAN							
	1996	1999	2002	2005	2008	2011	2014
Population (thousand person)	125,864	126,686	127,435	127,768	127,692	127,799	126,949
[% of 65 years or older]	15.1%	16.7%	18.5%	20.2%	22.1%	23.3%	26.1%
Number of deaths (person)	32,169	33,814	34,634	34,265	33,659	31,831	29,541
[% of 65 years or older]	63.1%	70.2%	75.5%	78.4%	81.3%	83.2%	86.9%
Number of incidence (person)	40,128	39,816	40,604	42,194	48,512	43,840	3667^※^
[% of 65 years or older]	62.8%	67.9%	70.8%	72.1%	76.7%	78.7%	79.2%^※^
Crude mortality/incidence rate	80.2%	84.9%	85.3%	81.2%	69.4%	72.6%	70.3%^※^
Average age of incidence (year)	67.5	68.5	69.5	70.1	71.5	72.6	72.8^※^
Average age of death (year)	67.9	69.0	70.5	71.9	73.2	74.5	75.8
Direct cost (billion yen) (billion USD)	103.7 (0.97)	122.2 (1.15)	156.2 (1.46)	147.6 (1.38)	140.7 (1.32)	138.4 (1.30)	131.6 (1.23)
Morbidity cost (billion yen) (billion USD)	28.7 (0.27)	35.0 (0.33)	34.0 (0.32)	31.5 (0.30)	26.4 (0.25)	21.4 (0.20)	18.5 (0.17)
Mortality cost (billion yen) (billion USD)	730.7 (6.85)	684.2 (6.41)	719.0 (6.74)	624.6 (5.86)	578.3 (5.42)	548.6 (5.14)	457.1 (4.29)
[% of 65 years or older]	27.0%	32.4%	42.1%	44.7%	48.1%	54.7%	58.5%
Mortality cost per person (million yen) (million USD)	22.7 (0.21)	20.2 (0.19)	20.8 (0.19)	18.2 (0.17)	17.2 (0.16)	17.2 (0.16)	15.5 (0.15)
COI (billion yen) (billion USD)	863.1 (8.09)	841.5 (7.89)	909.2 (8.52)	803.8 (7.54)	745.4 (6.99)	708.4 (6.64)	607.2 (5.69)
COI per population (yen) (USD)	6857.4 (64.29)	6642.2 (62.27)	7134.7 (66.89)	6291.2 (58.98)	5837.9 (54.73)	5543.0 (51.97)	4783.2 (44.84)
Source of population: Ministry of Internal Affairs and Communications “Population Estimates”							
Source of the number of cancer deaths: “Vital Statistics”							
Source of the number of incidence: Center for Cancer Control and Information Services, National Cancer Center, Japan.							
Average age of incidence: Calculated according to the number of incidence.							
Average age of death: Calculated according to the number of deaths, sex and age (5 years old age-grade), cause of death in “Vital Statistics”.							
※ 2012 data.							
TAIWAN							
	2002	2005	2008	2011	2014		
Population (thousand person)	22,521	22,770	23,037	23,225	23,417		
[% of 65 years or older]	8.9%	9.6%	10.3%	10.8%	11.9%		
Number of deaths (person)	6943	7108	7651	8022	8178		
[% of 65 years or older]	52.4%	55.5%	57.8%	59.5%	60.5%		
Number of incidence (person)	8860	9916	10,565	11,292	11,358		
[% of 65 years or older]	–	–	–	–	–		
Crude mortality/incidence rate	78.4%	71.7%	72.4%	71.0%	72.0%		
Average age of incidence (year)	–	–	–	–	–		
Average age of death (year)	63.6	64.6	65.7	67.0	67.8		
Direct cost (million NT$) (million USD)	4285.3 (135.72)	4392.7 (139.12)	6166.3 (195.30)	7038.6 (222.92)	8507.5 (269.45)		
Morbidity cost (million NT$) (million USD)	131.6 (4.17)	152.8 (4.84)	290.0 (9.18)	603.0 (19.10)	367.9 (11.65)		
Mortality cost (million NT$) (million USD)	17,713.3 (561.01)	16,719.4 (529.53)	17,636.7 (558.59)	16,962.1 (537.22)	16,879.4 (534.60)		
[% of 65 years or older]	14.0%	15.6%	16.3%	19.5%	19.8%		
Mortality cost per person (thousand NT$) (thousand USD)	2551.3 (80.80)	2352.2 (74.50)	2305.2 (73.01)	2114.5 (66.97)	2064.0 (65.37)		
COI (million NT$) (million USD)	22,130.2 (700.90)	21,264.9 (673.50)	24,093.0 (763.07)	24,603.7 (779.24)	25,754.8 (815.70)		
COI per population (NT$) (USD)	982.7 (31.12)	933.9 (29.58)	1045.8 (33.12)	1059.4 (33.55)	1099.8 (34.83)		
Source of population: Department of Household Resistration, Ministry of Interior							
Source of the number of cancer deaths: “Cause of Death Statistics,” Ministry of Health and Welfare							
Source of the number of incidence: “Health Statistics,” Ministry of Health and Welfare							
Average age of death: Calculated according to the number of deaths, sex and age (5 years old age-grade), cause of death in “Cause of Death Statistics”.							
※ 2012 data.							

The decreased MtC was the primary factor contributing to the decrease in the total COI in Japan. NDy decreased by 8.2% from 32,169 in 1996 to 29,541 in 2014. Moreover, the MtC per person continuously decreased by 31.7% from 22.7 million yen in 1996 to 15.5 million yen in 2014. Although DC decreased from 2002 to 2014, the unit cost increased; the hospital cost per day increased by 1.43 times, and the outpatient cost per visit increased by 2.15 times. However, the total DC decreased because of large decreases in the ALOS, number of inpatients, and number of outpatient visits. However, the increased DC was the primary factor contributing to the increase in the total COI in Taiwan. The unit hospital costs and inpatient costs increased by 1.96 times and 1.36 times, respectively. Moreover, the number of inpatients and outpatients increased. In contrast to Japan, the MtC in Taiwan was nearly stable due to an increase in NDy despite the decrease in MtC per person.

The decreases in the MtC per person in both countries were influenced by the increased average age of death. During the targeted period, the percentage of deaths among persons aged over 65 years increased from 63.1 to 86.9% in Japan, and the average age of death also increased from 67.9 years to 75.8 years. The percentage of deaths among persons aged over 65 years in Taiwan also increased from 52.4 to 60.5%, and the average age of death increased from 63.6 years to 67.8 years. The increased percentage of aged deaths decreased the MtC per person because the human capital value of elderly persons is lower than that of younger people. The ageing of the dead advanced more rapidly, which may explain the difference in the MtC trends between the two countries.

The MtC per person in both countries decreased because of an increase in the average age of death. Thus, the MtC in Japan remarkably decreased (APC: − 2.72%) due to the decrease in NDy, whereas the MtC in Taiwan slightly decreased (APC: − 0.44%), which was offset by the increase in NDy. The main factors affecting the change in NDy were the trends of the mortality rates of each age group and the ageing of society. In both countries, the older age groups had higher mortality rates, and this tendency was observed during the targeted period. The mortality rate of most age groups had a downward tendency; however, the mortality rate of the over 75 year group was stable in Japan. However, while the mortality rate of most age groups in Taiwan had a downward tendency (similar to Japan), the mortality rate of the over 75 year group had an upward tendency (from 209.9 per 100 thousand persons in 2002 to 226.8 per 100 thousand persons in 2014). In addition to the change in the mortality rate, the ageing of society had a great influence on NDy. The percentage of older age groups with higher mortality rates increased in both countries. Thus, the mortality rate effect in Japan outweighed the ageing effect, and the NDy decreased, whereas the mortality rate effect in Taiwan was outweighed by the ageing effect, and the NDy increased.

Regarding DC, the unit costs (hospital cost per day and outpatient cost per visit) increased in both countries; however, THD and TOVy increased in Taiwan, resulting in a continuous increase in DC, whereas THD and TOVy decreased in Japan, resulting in decreases in DC since 2002. The increases in THD, TOVy and NDy in Taiwan reflect increased incidence. The incidence of PLC in Japan has decreased since 2008; however, the incidence in Taiwan continued to increase until 2014.

**Fig. 1:**
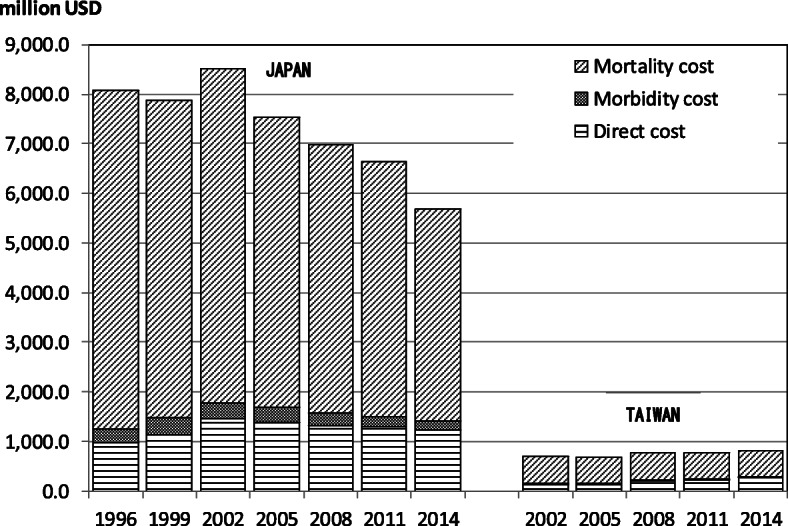
The trend of COI

**Fig. 2:**
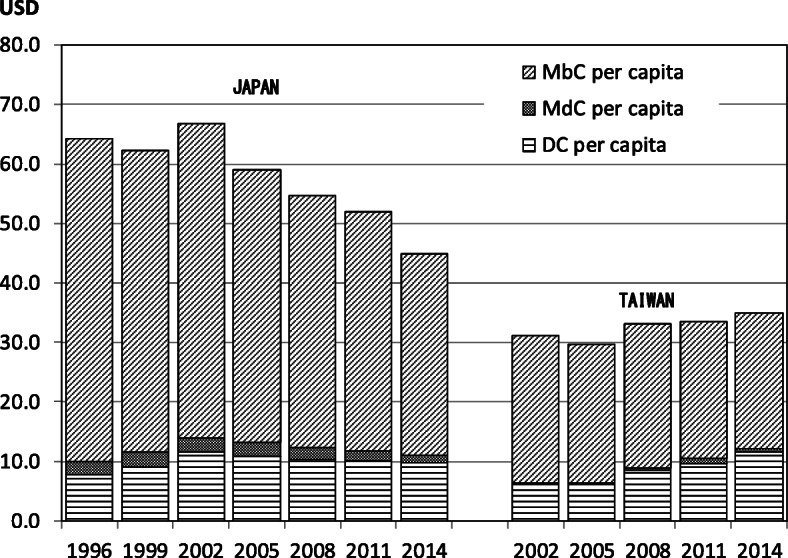
The trend of COI per capita

Fig. 1 and Fig. 2 show the COI trends in both countries. The data in both figures were converted to USD using the average exchange rate during the targeted periods to adjust for the influence of foreign exchange volatility. Figure 1 shows the total COI of PLC trends in both countries. Because of the differences in the population size, the Japanese COI was 7 times greater than that of Taiwan in 2014. Figure 2 shows the COI trends per capita, which had a downward tendency in Japan and an upward tendency in Taiwan. The COI per capita in Japan was 1.5 times higher than that in Taiwan.

## Discussion

COI of PLC per capita in both countries was 44.8 USD in Japan and 34.8 USD in Taiwan in 2014. It is necessary to be careful in comparing COIs per capita of both countries without considering the economic conditions and differences in medical systems of each country, but more important issue is the comparison of the COI trends of both countries. COI has been declining in Japan since 2002, while it has been increasing in Taiwan until 2014. This was considered to reflect the trends in the number of deaths and morbidity in both countries.

However, it is considered that there is no big difference in the treatment contents for PLC between the two countries. The treatment method for PLC differs depending on the situation and the degree of progression, but the 5-year survival rate has been shorter than that of other cancers. As for early stage cancer, liver transplantation was first treated in Taiwan in 1984 and Japan in 1986, respectively, but the proportion of liver transplantation for the treatment of early stage PLC is limited in both countries. Liver resection remains the primary treatment. As for advanced hepatocellular carcinoma, the result of randomized control trial of Sorafenib in the Asia-Pacific region was reported in 2009 [32], and the molecular target drug Sorafenib has been approved in both countries and has become the standard treatment [33]. In recent years, the effectiveness of immune checkpoint inhibitors such as Nivolumab has also been confirmed.

Given that there is no significant difference in PLC treatment between the two countries, the difference in the number of deaths and patients between the two countries may be due to the morbidity of viral hepatitis, which accounts for most of the causes of PLC. The decreased incidence of PLC in Japan was probably caused by health policies targeting HBV and HCV, accounting for 80% of patients with PLC [34]. Regarding HBV, in 1986, the Japanese government initiated a nationwide hepatitis B screening and immunization programme for infants born to HBV carrier mothers. The number of HBV carriers since the 1986 birth cohort has remarkably decreased. Anti-HCV screening of blood products has also been performed since 1989, and the use of nucleic acid technology to screen for HCV RNA was initiated in 1999. Medical treatments against hepatitis, such as interferon therapy, interferon + ribavirin therapy, and peg-interferon therapy, have also been developed [35]. Moreover, the appearance of DAA (direct acting antivirals) remarkably improved the therapeutic effect [36]. In Japan, DAAs, which are quite expensive, are covered by national health insurance, and accessibility to DAA is good. These treatments are effective, especially for HCV, and are considered responsible for the decreased PLC incidence since 2008.

The Taiwanese government also began a mass immunization programme among new-born babies against HBV in 1984. The programme was expanded to all people younger than 20 years in 1991. In addition, the national viral hepatitis therapy programme was launched in 2003 with noticeable effects [37–39].

The differences in the trends of PLC incidence despite such policies are attributed to the difference in the hepatitis types. PLC caused by chronic HCV infection is predominant in Japan, whereas HBV infection is the predominant cause of PLC in Taiwan. The therapeutic approach to HCV has recently rapidly progressed. Compared with HCV therapy, the therapeutic approach to HBV has slowly improved. This difference in progress is thought to be related to the different trends of PLC incidence between the two countries. However, the age-adjusted incidence in Taiwan has also decreased. Taiwan has already attained universal HBV vaccination, and similar to Japan, the number of HBV and PLC patients is expected to decrease.

This study was not without limitations. Firstly, COI is a simple and convenient approach that measures the burden of disease in terms of monetary cost, but nevertheless it is difficult to make a direct comparison between different countries. In this study, the COIs of both countries were displayed in US dollars using the average exchange rate during the observation period, but the USD terms of the COIs of both countries changes depending on which kind rate is used. In this study, we focused on what kind of trends COI is showing and whether there are differences in the proportions of costs that make up COI, rather than international comparisons on a monetary basis. Additionally, the COI method used here did not take the quality of the medical treatment provided or patients’ quality of life into consideration. Therefore, it does not examine the cost effectiveness of individual medical management. However, it is still useful because it enables comparison with COIs of other diseases and to regard the impacts of the aging population.

The COI of PLC in Japan has already remarkably decreased and is expected to decrease more rapidly in the future. PLC can be considered a successful case of health policy and technological development in Japan. The COI of PLC is expected to also decrease in Taiwan in the future.

## Conclusion

The findings of the present study suggest that the COI of PLC in Japan has continuously decreased to date, whereas that in Taiwan has continuously increased. Previous health policies and technological developments are considered to have accelerated the COI decrease in Japan and are expected to change the trend of COI of PLC in Taiwan.

## Data Availability

The datasets during and/or analysed during the current study available from the corresponding author on reasonable request.

## Abbreviations

COI: Cost of illness
PLC: Primary liver cancer
HBV: Hepatitis B virus
HCV: Hepatitis C virus
DC: Direct cost
IC: Indirect cost
MbC: Morbidity cost
MtC: Mortality cost
TOVy: Total person-days of outpatient visits
LVd: 1-day labour value per person
THD: Total person-days of hospitalization
Iy: Annual income per person
ULVy: Annual monetary valuation of unpaid work per person
HPy: Annual number of hospitalized patients
ALOS: Average length of stay
NDy: Number of deaths
LVl: Lifetime labour value per person
JPY: Japanese Yen
NTD: New Taiwan dollars
